# Desert dust outbreaks and respiratory morbidity in Athens, Greece

**DOI:** 10.1186/s12940-017-0281-x

**Published:** 2017-07-01

**Authors:** Stavroula-Myrto Trianti, Evangelia Samoli, Sophia Rodopoulou, Klea Katsouyanni, Spyros A. Papiris, Anna Karakatsani

**Affiliations:** 1 2nd Department of Pulmonary Medicine, School of Medicine, National and Kapodistrian University of Athens, “ATTIKON” University Hospital, Athens, Greece; 2 Department of Hygiene, Epidemiology and Medical Statistics, School of Medicine, National and Kapodistrian University of Athens, Athens, Greece

**Keywords:** Desert dust, Particulate matter, PM_10_, Respiratory morbidity

## Abstract

**Background:**

Ambient particulate matter (PM) has an adverse effect on respiratory morbidity. Desert dust outbreaks contribute to increased PM levels but the toxicity of desert dust mixed with anthropogenic pollutants needs clarification.

**Methods:**

We identified 132 days with desert dust episodes and 177 matched days by day of the week, season, temperature and humidity between 2001 and 2006 in Athens, Greece. We collected data on regulated pollutants and daily emergency outpatient visits and admissions for respiratory causes. We applied Poisson regression models adjusting for confounding effects of seasonality, meteorology, holidays and influenza epidemics. We evaluated the sensitivity of our results to co-pollutant exposures and effect modification by age and sex.

**Results:**

A 10 μg/m^3^ increase in PM_10_ concentration was associated with 1.95% (95% confidence interval (CI): 0.02%, 3.91%) increase in respiratory emergency room visits. No significant interaction with desert dust episodes was observed. Compared with non-dust days, there was a 47% (95% CI: 29%, 68%) increase in visits in dust days not adjusting for PM_10_. Desert dust days were associated with higher numbers of emergency room visits for asthma, chronic obstructive pulmonary disease and respiratory infections with increases of 38%, 57% and 60%, respectively (*p* < 0.001 for all comparisons). Analyses of respiratory hospital admissions provided similar results. PM_10_ effects decreased when adjusting for desert dust days and were further confounded by co-pollutants.

**Conclusions:**

Desert dust episode days are associated with higher respiratory emergency room visits and hospital admissions. This effect is insufficiently explained by increased PM_10_ levels.

**Electronic supplementary material:**

The online version of this article (doi:10.1186/s12940-017-0281-x) contains supplementary material, which is available to authorized users.

## Background

Transportation of desert dust, originating mainly from Sahara, is common in Europe, especially in the Mediterranean countries, resulting to increased concentrations of ambient particles with a median aerodynamic diameter < 10 μm (PM_10_) [1–4]. PM_10_ is a mixture of coarse (2.5-10 μm, PM_2.5-10_), fine (<2.5 μm, PM_2.5_) and ultrafine (<0.1 μm, PM_0.1_) particles generated from different processes, having variable chemical composition and atmospheric behavior. The adverse health effects of short-term exposures to PM_10_ and PM_2.5_ have been well documented during the last decades whilst from recent studies there is increasing evidence that the health effects of coarse particles should not be underestimated [5–8].

Recent evidence has shown that desert dust outbreaks alter particle size distribution as well as their chemical composition [9–12]. This effect depends on several factors such as the dust origin as well as the transportation route until the dust reaches the particular destination. Previous studies have indicated an increase in crustal elements of PM_2.5_ in dust days compared with non-dust days as well as differences in the concentrations of selected metals of larger particles [13]. Additionally, there is evidence for a microbial component of the transported dust [14]. However, from the limited number of studies carried out to establish the health effects due to the desert dust source, there are conflicting results [15].

The Athens area in Greece, with a population over 4 million inhabitants, faces a serious air pollution problem that has existed for more than 30 years. Its topography, a basin surrounded by mountains in the north, east and northwest and by the sea on the southwest side, favours atmospheric inversion and high pollutants’ concentrations. Previous studies have shown effects of air pollution on respiratory mortality and morbidity in the Greater Athens area [16–18].

Desert dust events generally occur about 30 days per year in Athens, mainly between spring and autumn [19]. Most such events originate from Sahara but there are a few days when they originate from the Arabian Peninsula. Previous studies on the health effects of desert dust episodes in Athens indicated that effects on mortality outcomes [20] could not be sufficiently captured by PM levels while they were associated with an increase in pediatric asthma admissions [21].

The rather limited evidence warrants further investigation as determining the possible effects of exposure to desert dust particles may be directly linked to policy decisions. In the present study, to further clarify the potential toxicity of desert dust mixed with anthropogenic pollutants, we investigated the effect of desert dust events on respiratory morbidity in the Southern Mediterranean metropolitan region of Athens, Greece.

## Methods

Between 2001 and 2006, we identified 132 days with desert dust episodes, in Athens, Greece, using back-trajectory analysis (to locate air mass transport) in combination with a data driven criterion, based on high particle concentrations provided by the fixed monitoring sites [20]. In brief, a day was defined as one with dust event if air mass transport, identified through the back-trajectories maps, had occurred from the Sahara or the Arabian Peninsula and the ratio of the PM_10_ concentration measured at a suburban monitor located at the outskirts of the Athens north region to an urban monitor in the center of the town exceeded the median of this ratio during that year.

During the same time period, we selected 177 control days fulfilling the following criteria: same day of the week and same season with the desert dust day as well as similar meteorological data, i.e. the desert dust and control day should not differ in mean temperature and humidity by no more than 2^ο^C and 7%, respectively. Same season was defined as the same season within the same calendar year with exception of the winter which extended from December of a given year to February of the next year.

Daily air pollution concentrations were provided by the monitoring network operated by the Ministry of Environment, Energy and Climate Change (www.ypeka.gr): for PM_10_ and NO_2_ daily 24 h average data, for O_3_ daily 8 h maximum data.

Daily data of emergency outpatient visits and admissions for respiratory causes of adult patients were recorded from 1st January 2001 to 31th December 2006 for the days that were identified as days with desert dust events as well as for the control days in 16 out of 19 pulmonary departments (84%) participating in the emergency services network of the Athens’ Metropolitan Area. The collection of data was performed retrospectively by reviewing the respective hospital records that provide a complete and consecutive registration of all emergency room visits and admissions. Exclusion criteria included: a) scheduled hospital admissions during the specific days, b) emergency room visits of patients who were transferred to hospitals outside the larger Athens area (Attica) or who were not residents of Athens, c) emergency room visits, for which the symptoms were attributed to tuberculosis, neoplastic diseases, aspiration pneumonia, accidental inhalation of smoke or toxic gases, fever or chest pain of non-respiratory origin and cases with prolonged symptoms. In all registered cases, the following parameters were recorded: date of visit, age, gender, final diagnosis, and outcome defined as discharge directly from the emergency room, hospital admission, or death in the emergency room.

Due to the lack of electronic records for emergency room visits and relevant data, such as demographics, diagnostics, performed examinations and outcome (i.e. discharge or admission, recommendations to the patient etc), all data analyzed for the present work were collected manually by reviewing the emergency department books kept in the hospitals’ archives for the a-priori defined desert dusts days and their matched control days during the study period.

Medical ethical clearance was acquired from the institutional review boards of all involved hospitals.

We applied Poisson regression models adjusting for any remaining confounding effects of seasonality and long term trends using a two way interaction between year and month. For both PM_10_ and for dust events we used lag 0 as an a-priori choice. We controlled for meteorology using a natural spline with 3 degrees of freedom for mean daily temperature and a linear term for relative humidity. We also included indicator variables for day of the week, holidays and influenza epidemics.

We evaluated the sensitivity of our results to co-pollutant exposures and effect modification by age (below and above 65 years) and sex. We furthermore excluded events for which we estimated the age in the age stratified analysis. Finally, we assessed robustness by using the approach adopted by the MED-PARTICLES project to characterize a day as desert dust or not (within the sample of days with available health data in our analysis) [22]. In brief, MED-PARTICLES investigators have used a variety of tools (meteorological products, aerosol maps, back trajectories, satellite images and the reference to identify dust transport and supplied us with their characterization upon our request.

As additional sensitivity analyses, we applied two modelling approaches that preserved the matching of “dust” and “non-dust” days but use more degrees of freedom in the models. In the first approach, we added 132 indicators for the matched sets and in the second approach, we applied a mixed Poisson model with a random intercept for matched sets of days.

## Results

The distribution of daily meteorological and air pollution data as well as the daily number of emergency outpatient visits and admissions for respiratory causes stratified by desert dust and matching control days are presented in Table 1.

**Table 1:** Distribution of pollution and meteorological data and daily respiratory emergency room visits and hospital admissions during dust and control days as well as pollution and meteorological data for the whole 6-year period

	Desert dust days *N* = 132	Control days *N* = 177	Whole period
			2001-2006
Pollution data in μg/m^3^ (median (25th–75th percentile))			
PM_10_	45.9 (36.5–78.5)	44.0 (34.6–54.2)	39.6 (30.7–50.8)
SO_2_	10.3 (6.8–16.4)	13.8 (9.0–20.3)	13.0 (7.9–20.6)
NO_2_	40.3 (32.0–48.9)	48.0 (39.7–60.4)	44.2 (34.8–54.6)
O_3_	61.8 (46.5–72.4)	71.7 (51.4–90.9)	68.7 (47.5–90.2)
Meteorological data (mean (standard deviation))			
Temperature (°C)	18.9 (5.2)	19.1 (5.9)	18.0 (12.4–24.8)
Relative humidity (%)	69.1 (14.8)	67.1 (14.4)	67.1 (56.0–76.9)
Number of daily emergency room visits (median (25th–75th percentile))			
Total visits per day	50 (35–68)	38 (25–47)	
Male patients	27 (19–35)	19 (12–25)	
Female patients	23 (15–32)	17 (12–23)	
Age 18-64 years	30 (19–39)	22 (14–27)	
Age ≥ 65 years	20 (13–29)	16 (10–21)	
Total hospital admissions per day	15 (10–22)	12 (6–16)	
Male patients	8 (5–13)	7 (4–10)	
Female patients	5 (3–9)	4 (2–7)	
Age 18-64 years	5 (3–8)	4 (2–6)	
Age ≥ 65 years	9 (6–14)	8 (4–11)	

The distribution of days with desert dust events by season and year, presented in Table 2, reveals that dust episodes tend to occur on spring (31.1% of dust days) or fall (28% of dust days) that is periods with lower pollution in general. Over the 6 years of the examined period, there were differences in the yearly distribution with highest occurrence of events in 2001, 2002 and 2004 and considerably less dust events in the other years.

**Table 2:** Distribution of days (number and percent) with desert dust events by season and year

Season	Winter	33 (25.0)
	Spring	41 (31.1)
	Summer	21 (15.9)
	Fall	37 (28.0)
Year	2001	32 (24.2)
	2002	31 (23.5)
	2003	15 (11.4)
	2004	26 (19.7)
	2005	13 (9.9)
	2006	15 (11.4)

During the study period, a total of 13,685 emergency room visits (47% females) were recorded. Out of these, 4213 patients were admitted for further management whilst 10 died in the emergency room. For 4 patients, information on the outcome was missing. For 1929 visits (14%) the patient’s age was not available, these missing values were replaced by the mean age of patients with the same diagnosis.

Table 3 presents the estimated effects of desert dust events and PM_10_ concentrations on the numbers of emergency room visits for respiratory causes, stratified by gender, age group (below or above 65 years) and diagnoses (asthma, chronic obstructive pulmonary disease [COPD], respiratory infections). The effect estimates of either dust episodes or PM_10_ were statistically significant when individually introduced in the model. There was a 47% (95% confidence interval (CI) 28.65%-68.19%; *p* < 0.001) increase in the total number of emergency room visits during desert dust versus non-dust days, not adjusting for PM_10_. No effect modification was observed by gender or age group. With respect to specific respiratory diagnoses, desert dust days were associated with higher numbers of emergency room visits for asthma, COPD and respiratory infections with increases of 38, 57 and 60%, respectively (for all, *p* < 0.001). Furthermore, a 10 μg/m^3^ increase in the PM_10_ concentration was associated with 1.95% (95% CI: 0.02%, 3.91%; *p* = 0.05) increase in total respiratory emergency room visits, not adjusting for desert dust events or other air pollutants. This effect was more pronounced among males and in patients older than 65 years [2.2% (*p* = 0.03) and 2.1% (*p* = 0.05) increase for a 10 μg/m^3^ PM_10_ increase, respectively). As for specific respiratory diagnoses, PM_10_ concentrations were significantly associated with increased numbers of emergency room visits for COPD but not for asthma or respiratory infections.

**Table 3:** Percent increase and 95% confidence interval (in parentheses) of daily respiratory emergency room visits associated with 10 μg/m^3^ increase in PM_10_ levels and occurrence of desert dust event

Emergency Room Visits	Individually in the model				Mutually Adjusted			
	PM_10_ (per 10 μg/m^3^)		Dust Day (Yes vs No)		PM_10_ (per 10 μg/m^3^)		Dust Day (Yes vs No)	
	% (95% CI)	*P*-value	% (95% CI)	*P*-value	% (95% CI)	*P*-value	% (95% CI)	*P*-value
All respiratory	1.95 (0.02,3.91)	0.05	47.09 (28.65,68.19)	< 0.001	0.99 (−0.88, 2.89)	0.30	45.25 (26.75,66.45)	< 0.001
Male	2.17 (0.18,4.2)	0.03	45.91 (26.7,68.03)	<0.001	1.25 (−0.7,3.24)	0.21	43.60 (24.42,65.73)	<0.001
Female	1.81 (−0.26,3.93)	0.09	45.81 (26.38,68.24)	<0.001	0.86 (−1.16,2.91)	0.41	44.22 (24.66,66.86)	<0.001
18–64 years	1.86 (−0.16,3.92)	0.07	49.09 (29.64,71.45)	<0.001	0.88 (−1.08,2.88)	0.38	47.46 (27.93,69.99)	<0.001
≥ 65 years	2.07 (−0.01,4.19)	0.05	44.34 (24.65,67.13)	<0.001	1.14 (−0.89,3.22)	0.27	42.17 (22.46,65.07)	<0.001
Asthma (J45)	1.98 (−0.41,4.42)	0.11	38.37 (16.84,63.85)	<0.001	1.17 (−1.21,3.61)	0.34	36.33 (14.79,61.91)	<0.001
COPD (J44)	3.21 (0.9,5.58)	0.007	57.14 (33.22,85.35)	<0.001	2.11 (−0.15,4.42)	0.07	52.91 (29.35,80.75)	<0.001
Respiratory infections (J06)	1.17 (−1.32,3.71)	0.36	60.43 (35.37,90.13)	<0.001	−0.03 (−2.45,2.45)	0.98	60.49 (35.02,90.77)	<0.001

*CI* confidence interval. In parentheses, the ICD (International Statistical Classification of Diseases and Related Health Problems) 10 codes are given

The estimated adverse desert dust effects on respiratory morbidity were robust to adjustment to PM_10_, while particles’ effects were confounded by the inclusion of the dust indicator but retaining the adverse association (Table 3).

Table 4 presents the percent increase and 95% CI of daily respiratory hospital admissions associated with desert dust events and a 10 μg/m^3^ increase in PM_10_ concentrations. The pattern observed in all outcomes analyzed was the same as the one observed for daily emergency room visits during desert dust episodes versus non desert dust days. Similarly to the effect on emergency room visits, the particles’ effects retained their adverse association when the dust indicator was included in the model. This pattern was observed in both male and female patients as well in younger and older patients.

**Table 4:** Percent increase and 95% confidence intervals (CI) of daily numbers of admissions (including deaths) due to respiratory emergency visits associated with 10 μg/m^3^ increase in PM_10_ levels and occurrence of desert dust event

Admissions	Individually in the model				Mutually Adjusted			
	PM_10_ (per 10 μg/m^3^)		Dust Day (Yes vs No)		PM_10_ (per 10 μg/m^3^)		Dust Day (Yes vs No)	
	% (95% CI)	*P*-value	% (95% CI)	*P*-value	% (95% CI)	*P*-value	% (95% CI)	*P*-value
All respiratory	1.60 (−0.69,3.95)	0.17	40.68 (20.23,64.61)	< 0.001	0.70 (−1.57,3.03)	0.55	39.44 (18.82,63.64)	< 0.001
Male	1.92 (−0.58,4.47)	0.14	41.71 (19.09,68.63)	<0.001	1.04 (−1.46,3.59)	0.42	39.9 (17.22,66.98)	<0.001
Female	1.26 (−1.33,3.93)	0.34	38.52 (16.03,65.37)	<0.001	0.36 (−2.23,3.01)	0.79	37.87 (15.05,65.21)	<0.001
18 - 64 years	1.63 (−1.3,4.65)	0.28	41.1 (15.57,72.27)	0.001	0.70 (−2.25,3.73)	0.65	39.87 (14.13,71.43)	<0.001
> 65 years	1.59 (−0.75,3.99)	0.19	40.39 (19.35,65.14)	<0.001	0.71 (−1.62,3.09)	0.55	39.13 (17.92,64.17)	<0.001

We also allowed for an interaction term between PM_10_ and desert dust events in our models. There was not statistically significant interaction for any of the associations under investigation.

As shown in Table 5, our estimates were robust to the alternative inclusion of NO_2_ or O_3_ into our model. The effect estimate of desert dust events on all respiratory emergency room visits or on hospital admissions remained highly significant (*p* < 0.001 for all analyses) whereas the PM_10_ concentrations had no significant effect on these health outcomes in the mutually adjusted model. Additional file 1: Table S1 provides the effect estimates for the association between gaseous pollutants and respiratory morbidity.

**Table 5:** Percent increase and 95% confidence intervals (CI) of daily respiratory emergency room visits and hospital admissions associated with 10 μg/m^3^ increase in PM_10_ levels and occurrence of desert dust event. Results from two pollutants’ models

	Individually in the model		Mutually Adjusted	
Respiratory Health Outcomes	% (95% CI)	*P*-value	% (95% CI)	*P*-value
All emergency room visits				
Adjusted for NO_2_				
PM_10_ (per 10 μg/m^3^)	1.93 (0.065, 3.84)	0.04	1.04 (−0.83, 2.94)	0.28
Dust Day (Yes vs No)	44.78 (25.34, 67.25)	<0.001	42.50 (23.00, 65.09)	<0.001
Adjusted for O_3_				
PM_10_ (per 10 μg/m^3^)	2.03 (0.09, 4.01)	0.04	1.15 (−0.71, 3.05)	0.23
Dust Day (Yes vs No)	50.20 (31.09, 72.10)	<0.001	48.25 (29.18, 70.14)	<0.001
All admissions				
Adjusted for NO_2_				
PM_10_ (per 10 μg/m^3^)	1.62 (−0.62, 3.91)	0.16	0.75 (−1.54, 3.08)	0.53
Dust Day (Yes vs No)	39.19 (17.40, 65.04)	<0.001	37.59 (15.78, 63.79)	<0.001
Adjusted for O_3_				
PM_10_ (per 10 μg/m^3^)	1.73 (−0.58, 4.09)	0.14	0.90 (−1.37, 3.22)	0.44
Dust Day (Yes vs No)	43.90 (22.69, 68.78)	<0.001	42.45 (21.18, 67.45)	<0.001

After applying the criteria used in the MED-PARTICLES project to redefine days as either “a desert dust day” or “not a desert dust day”, the days with common characterization we ended up, were 81 dust days and 110 control (non-dust) days. In sensitivity analyses, the direction as well as the magnitude of the effects remained robust when we included only the days with common characterization based on both approaches (Additional file 1: Table S2).

Results from the sensitivity analysis in the subgroup of patients with complete information on their age provided similar results both in the magnitude of the effects and in the statistical significance. For example, the percent change in emergency room visits for respiratory causes in the mutually adjusted model for those 18-64 years was 0.53% (95% CI: -1.48%, 2.58%) per 10 μg/m^3^ increase in PM_10_ and 53.20% (95% CI: 32.56%, 77.06%) for the dust indicator; for those **≥**65 years the corresponding estimates were 0.51% (95% CI: -1.67%, 2.74%) and 46.19% (95% CI: 24.88%, 71.14%). The results from the additional sensitivity analysis models applied did not substantially change the main findings (Additional file 1: Tables S3 and S4).

## Discussion

Our results support that desert dust transportation affects respiratory morbidity in Athens, Greece, as we observed associated increases in respiratory emergency room visits and hospital admissions for respiratory reasons. Interestingly, this effect does not seem to be at least sufficiently explained by increased PM_10_ levels.

Desert dust outbreaks are frequent over the Mediterranean basin and may be directly linked to policy decisions as they contribute significantly to the burden of atmospheric mineral dust and to PM_10_ concentrations exceeding the daily recommended limits [1–4, 19, 23]. Desert dust consists of coarse particles which have been associated mainly with respiratory outcomes. Recent epidemiological and toxicological studies indicate that the health effects of coarse particles should not be underestimated and that their levels should be regulated along with those of fine particles [7, 8, 24].

Thus, for reasons of public health protection, it is of great importance to clarify potential adverse health effects of desert dust outbreaks. So far, studies on the health effects of such outbreaks or on their modifying role in particulate matter health effects are limited and have reported contradictory results [15].

Studies focusing on the effect of desert dust outbreaks on mortality that were conducted in the Mediterranean and particularly in Spain [13, 25] and Italy [26, 27] provided evidence for an independent association between such events and daily mortality. They indicated a stronger effect of coarse particles and PM_10_ on mortality during dust days but also an increased effect on the elderly [28]. In contrast, Samoli et al. [20] did not report effect modification of PM_10_ effects on mortality in Athens, Greece by desert dust. Similarly, in a project conducted in multiple Mediterranean areas, PM_10_ concentrations originating from desert dust and those from other sources were both associated with mortality [22].

In our study, we focused on the effect of desert dust outbreaks and on the potential modifying effect on PM_10_ effects particularly on *respiratory morbidity*. We consider the focus on respiratory morbidity a major advantage of our study due to the plausible biological explanation. The respiratory system belongs to the systems of the human body, for which there is convincing evidence for a significant influence exerted by meteorological and atmospheric conditions and an associated effect on morbidity [29–31]. Thus, it seems plausible that adverse effects of an increased pollution burden and of altered atmospheric conditions due to desert dust outbreaks would manifest predominantly in the respiratory system. Indeed, several mechanisms for an adverse effect of particulate matter on the respiratory system have been proposed including for instance local inflammatory reaction [32], the induction of a systemic inflammatory response augmenting lung inflammation [33], or cytotoxicity and oxidative damage [34, 35]. Furthermore and to the best of our knowledge, this is the first study that addresses the effects of desert dust events on respiratory emergency room visits apart from admissions and also investigates associations with *specific* respiratory diseases.

Our results are in general consistent with other morbidity studies from the Mediterranean. Middleton et al. observed an increased hospitalization risk particularly for cardiovascular causes on dust storm days in Cyprus [36]. Similarly, Samoli et al. reported more emergency hospital admissions on pediatric asthma during Sahara dust events, in Athens, Greece [21]. There are also data on strong effects of coarse particles on respiratory and of PM_10_ on cerebrovascular diseases during desert dust outbreaks [37]. Consistent with these results are also the findings of the MED-PARTICLES project that investigated the mortality and the cardiovascular and respiratory hospital admissions in multiple Mediterranean areas between 2001 and 2010. Increases in PM_10_ were positively associated with increases in mortality and hospital admissions due to cardio-respiratory causes [38]. Thus, in general, the results of morbidity studies provide more consistent indication of associations compared to those investigating the desert dust effects on mortality.

**Fig. 1:**
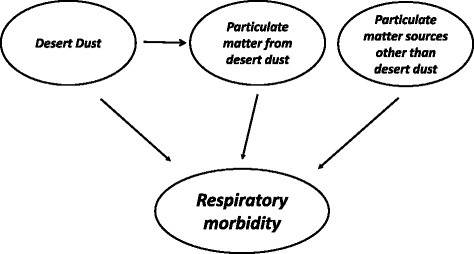
Directed acyclic graph for the suggested associations between desert dust events, PM_10_ levels and respiratory morbidity. According to the suggested underlying mechanisms, particulate matter has a direct effect on respiratory morbidity. Dust events contribute to the levels of particulate matter and thus affect respiratory morbidity but may also have an effect on respiratory morbidity by other mechanisms independent of the levels of particulate matter

The effects of dust could be attributed to a complex mixture of factors, including the change in the concentration and profile of particles, the lowering of the layer boundary height or/and the transport of pollen or bacteria. We hypothesized that although the greater part of desert dust effects may be attributed to particles’ effects there may also be other mechanisms, as for example the lowering of the boundary height may create a more toxic air pollution profile multiplying the effects of various air pollutants. Unfortunately we have no data to test any of these hypotheses. Hence, we consider the dust as an effect modifier for PM mass health effects, and PM separated in two components: one as a mediator of dust effects and another mainly characterized by PM from local emissions as an independent risk factor. However we only have one measurement for the total mass of PM and we can only approach the 2 components by testing the interaction of PM with desert dust days. The proposed associations are schematically depicted in Fig. 1. Unfortunately, lack of data for pollen or particles’ components data in the Athens’ region for the study period does not allow us to perform further sensitivity analyses.

Our finding of effects on respiratory morbidity during desert dust events, after adjusting for PM_10_, corroborates the hypothesis that these effects maybe partly attributed to specific biogenic factors and particles’ chemical constituents transferred from Africa not captured by measured particles’ concentrations [39]. PM_10_ are a heterogeneous group containing particles of both human and natural sources, hence depending on their chemical composition they may have different toxicity. Thus, apart from the adverse health effect of the particles, desert dust has further characteristics that may result in additional adverse health outcomes. These may be the conveyed metals, microorganisms or other components. The additional toxicity of this mixture that forms the desert dust particles may not be assessed by the global measurement of particles’ mass. Further meteorological factors such as wind direction, duration and speed may also modify health outcomes [40]. The exact geographic setting may also play a role: during the course of the dust clouds, the composition of the dust changes due to enrichment with additional elements leading to different chemical mixture and thus health effects [15]. Although these factors seem to provide a reasonable explanation for our findings, this remains speculative considering that some studies observed no or only moderate association of chemical composition with the health effects [13, 41].

The PM_10_ levels in our study showed no relevant difference in days with desert dust outbreaks compared with days without dust outbreaks. This is surprising given the fact that desert dust by itself conveys particulate matter. The only reasonable explanation for this would be a decreased level of particles from human sources in dust days resulting in similar total levels of particles. The selection of control days with the criterion of similar meteorological data may have contributed to this although this remains speculative.

A limitation of our study is that we could not analyze particulate fractions individually, since PM_2.5_concentrations were not measured in Athens during the analysis period. However, our results confirm the adverse effects of PM_10_ from all sources on respiratory morbidity previously identified. Another major limitation is the small sample size that is however partly compensated by the large underlying population and number of counts. This is partly due to our inability to extend the analysis to a longer period because of the lack of electronic records for emergency room visits and of the relevant data for the study periods. This lack of the required IT infrastructure renders an extension of the analysis to a longer time period practically impossible restricting the relevance of our findings.

The observed association of desert dust days and respiratory morbidity was very strong with estimates of approx. 50% increase in risk of emergency room visits or admissions on dust days compared with the non-dust days. These effects although consistent across all tested strata such as male/female or age below/above 65 years as well as across the different outcomes, are much larger than the effects usually reported in the literature. For this reason, we cannot exclude the possibility of uncontrolled confounding or of a chance finding. However, applying sensitivity analysis, did not modify the magnitude of effects, which remain large.

## Conclusions

In conclusion, days with desert dust transportation were associated with significantly increased emergency room visits and admissions for respiratory disorders such as asthma, COPD and respiratory infections in the metropolitan region of Athens, Greece. This effect was independent of PM_10_ concentrations and was of a clinically significant magnitude with increases of the different outcomes by 40-50%.

## Additional file

Additional file 1: Supplemental Tables. (DOCX 20 kb)

## Abbreviations

CI: Confidence interval
COPD: Chronic obstructive pulmonary disease
ICD: International Statistical Classification of Diseases and Related Health Problems
PM: Particulate matter
