# Supplementary material for: Desert dust outbreaks and respiratory morbidity in Athens, Greece
Source: Environ Health. 2017 Jul 1;16:72. doi: 10.1186/s12940-017-0281-x (PMC5493869; doi:10.1186/s12940-017-0281-x)
Supplement: Additional file 1: — Supplemental Tables. (DOCX 20 kb) [file 12940_2017_281_MOESM1_ESM.docx]

**Supplemental Table S1**

Percent increase and 95% confidence intervals (CI) of daily respiratory emergency room visits and hospital admissions associated with 10 μg/m^3^ increase in NO_2_ and O_3_ levels, respectively.

|  | **Individually in the model** | | **Controlling for dust days** | |
| --- | --- | --- | --- | --- |
| **Respiratory Health Outcomes** | **% (95% CI)** | **P-value** | **% (95% CI)** | **P-value** |
| **All emergency room visits** |  |  |  |  |
| NO_2_ (per 10 μg/m^3^) | -6.75 (-11.68, -1.55) | 0.01 | -1.66 (-6.95, 3.93) | 0.55 |
| O_3_ (per 10 μg/m^3^) | 0.77 (-3.73, 5.48) | 0.74 | 3.55 (-0.89, 8.18) | 0.12 |
|  |  |  |  |  |
| **All admissions** |  |  |  |  |
| NO_2_ (per 10 μg/m^3^) | -5.85 (-11.43, 0.08) | 0.05 | -1.08 (-7.23, 5.48) | 0.74 |
| O_3_ (per 10 μg/m^3^) | 1.49 (-3.60, 6.84) | 0.57 | 3.95 (-1.18, 9.35) | 0.14 |

**Supplemental Table S2**

Percent increase and 95% confidence interval (in parentheses) of a) daily respiratory emergency room visits and b) admissions (including deaths) due to respiratory emergency visits associated with 10 μg/m^3^ increase in PM_10_ levels and occurrence of desert dust event *with desert dust day definition according to the criteria used in the MED-PARTICLES project [22]*

|  | **Individually in the model** | | | | **Mutually Adjusted** | | | |
| --- | --- | --- | --- | --- | --- | --- | --- | --- |
|  | **PM_10_ (per 10μg/m^3^)** | | **Dust Day (Yes vs No)** | | **PM_10_ (per 10μg/m^3^)** | | **Dust Day (Yes vs No)** | |
|  | **% (95% CI)** | **P-value** | **% (95% CI)** | **P-value** | **% (95% CI)** | **P-value** | **% (95% CI)** | **P-value** |
| **Respiratory emergency room visits** | 1.59 (-0.78, 4.01) | 0.19 | 61.92 (32.67, 97.62) | < 0.001 | 0.37 (-1.91, 2.71) | 0.75 | 60.80 (31.01, 97.36) | < 0.001 |
| **Admissions** | 1.50 (-1.24, 4.31) | 0.29 | 45.73 (15.41, 84.02) | 0.002 | 0.52 (-2.25, 3.36) | 0.72 | 44.34 (13.52, 83.55) | 0.003 |

| **Emergency Room Visits** | **Individually in the model** | | | | **Mutually Adjusted** | | | |
| --- | --- | --- | --- | --- | --- | --- | --- | --- |
|  | **PM_10_ (per 10μg/m^3^)** | | **Dust Day (Yes vs No)** | | **PM_10_ (per 10μg/m^3^)** | | **Dust Day (Yes vs No)** | |
|  | **% (95% CI)** | **P-value** | **% (95% CI)** | **P-value** | **% (95% CI)** | **P-value** | **% (95% CI)** | **P-value** |
| **All respiratory (original model)** | 1.95 (0.02,3.91) | 0.05 | 47.09 (28.65,68.19) | < 0.001 | 0.99 (-0.88, 2.89) | 0.30 | 45.25 (26.75,66.45) | < 0.001 |
| **All respiratory (model with indicators for sets of matched "dust" and "non-dust" days)** | 1.61 (-0.47, 3.74) | 0.17 | 44.00 (26.96, 63.33) | < 0.001 | 0.26 (-1.74, 2.30) | 0.80 | 43.43 (25.94, 63.36) | < 0.001 |
| **All respiratory (mixed Poisson model with random intercept for sets of matched "dust" and "non-dust" days)** | 1.56 (0.93, 2.18) | < 0.001 | 42.05 (36.22,48.13) | <0.001 | 0.16 (-0.49,0.81) | 0.64 | 41.66 (35.63,47.95) | <0.001 |

**Supplemental Table S3**: Percent increase (and 95% confidence intervals) of daily respiratory emergency room visits associated with 10 μg/m^3^ increase in PM_10_ levels and occurrence of desert dust event, using 3 different modeling approaches.

| **Emergency Room Admissions** | **Individually in the model** | | | | **Mutually Adjusted** | | | |
| --- | --- | --- | --- | --- | --- | --- | --- | --- |
|  | **PM_10_ (per 10μg/m^3^)** | | **Dust Day (Yes vs No)** | | **PM_10_ (per 10μg/m^3^)** | | **Dust Day (Yes vs No)** | |
|  | **% (95% CI)** | **P-value** | **% (95% CI)** | **P-value** | **% (95% CI)** | **P-value** | **% (95% CI)** | **P-value** |
| **All respiratory (original model)** | 1.60 (-0.69,3.95) | 0.17 | 40.68 (20.23,64.61) | < 0.001 | 0.70 (-1.57,3.03) | 0.55 | 39.44 (18.82,63.64) | < 0.001 |
| **All respiratory (model with indicators for sets of matched "dust" and "non-dust" days)** | 1.81 (-0.60,4.29) | 0.14 | 33.68 (14.99, 55.42) | < 0.001 | 0.69 (-1.74, 3.19) | 0.58 | 32.21 (13.11, 54.54) | 0.001 |
| **All respiratory (mixed Poisson model with random intercept for sets of matched "dust" and "non-dust" days)** | 1.37 (0.27, 2.48) | 0.014 | 31.54 (22.11,41.69) | <0.001 | 0.20 (-0.95,1,36) | 0.74 | 31.03 (21.24,41.62) | <0.001 |

**Supplemental Table S4:** Percent increase (and 95% confidence intervals) of daily numbers of admissions (including deaths) due to respiratory emergency visits associated with 10 μg/m^3^ increase in PM_10_ levels and occurrence of desert dust event using 3 different modeling approaches
